# Non-prescribed use of methadone and buprenorphine prior to opioid substitution treatment: lifetime prevalence, motives, and drug sources among people with opioid dependence in five Swedish cities

**DOI:** 10.1186/s12954-019-0301-y

**Published:** 2019-05-02

**Authors:** Björn Johnson, Torkel Richert

**Affiliations:** Department of Social Work, Malmö University, Malmö, Sweden

**Keywords:** Opioid substitution treatment, Methadone, Buprenorphine, Buprenorphine/naloxone, Non-prescribed use, Pseudo-therapeutic use, Diversion, Opioid dependence

## Abstract

**Background:**

Opioid substitution treatment (OST) with methadone or buprenorphine is the most effective means of treating opioid dependence. If these substances are used by people who are not undergoing OST, they can however carry serious risks. This article examines the lifetime prevalence, motives, and drug sources for such use, as well as geographical differences in these variables.

**Methods:**

Structured interviews were conducted with 411 patients from 11 OST clinics in five Swedish cities. The researchers carried out 280 interviews on-site, while 131 interviews were conducted by specially trained patients through privileged access interviewing. Data were analyzed by frequency and average calculations, cross-tabulations, and *χ*^2^ tests.

**Results:**

The lifetime prevalence of non-prescribed use was 87.8% for methadone, 80.5% for buprenorphine, and 50.6% for buprenorphine/naloxone. Pseudo-therapeutic motives—avoiding withdrawal symptoms, staying clean from heroin, detoxification, or taking care of one’s own OST—were commonly cited as driving the use, while using the drugs for euphoric purposes was a less common motive. Most respondents had bought or received the substances from patients in OST, but dealers were also a significant source of non-prescribed methadone and buprenorphine. Geographical differences of use, motives, and sources suggest that prescription practices in OST have a great impact on which substances are used outside of the treatment.

**Conclusions:**

Experiences of non-prescribed use of methadone and buprenorphine are extremely common among those in OST in southern Sweden. As the use is typically driven by pseudo-therapeutic motives, increased access to OST might decrease the illicit demand for these substances. Buprenorphine/naloxone has a lower abuse potential than buprenorphine and should therefore be prioritized as the prescribed drug. Supervised dosage and other control measures are important provisions in the prevention of drug diversion and non-prescribed use among people not undergoing OST.

## Background and aims

Opioid substitution treatment (OST) with methadone or buprenorphine (including buprenorphine/naloxone) is considered the most effective means of treating opioid dependence. There is ample research evidence of positive outcomes in terms of reduced mortality, morbidity, illicit drug use, and criminality [1–4].

Methadone and buprenorphine are safe and effective when used according to the prescription [5, 6]. However, if they are used improperly (in a way contravening the prescription) or by people who are not undergoing OST, these substances can carry serious risks. There is a high risk of developing dependence, and overdosing may lead to death as a result of depressed ventilation. If the substances are injected, the risks are similar to those of injected heroin, including overdose, life-threatening infections, and transmission of blood-borne diseases [7, 8].

Methadone-related deaths have been highlighted as a problem in many countries [5, 9–12]. Buprenorphine, too, has been involved in many deaths, mainly in polydrug poisonings with sedative substances and alcohol [13–15]. Those who have died with these substances found in their body have mainly acquired the substances illicitly, that is, they have not themselves been in OST [12, 16–18].

And yet, the non-prescribed use of methadone and buprenorphine can also bring benefits. According to Harris and Rhodes, who have studied the non-prescribed use of methadone, such use can serve as a “protective strategy” and can enable opioid-dependent persons to control their drug use, improve their social relations, and reduce the transmission of blood-borne viruses as a result of decreased injecting [19]. Non-prescribed use of buprenorphine has also shown to carry fewer health risks and lead to a better quality of life than the use of heroin [6, 20].

This article examines experiences of previous, non-prescribed use of methadone and buprenorphine in a group of 411 persons in OST in 5 cities in southern Sweden. The data was collected in 2012 from structured interviews in conjunction with a research project focusing on drug diversion and non-prescribed use of methadone and buprenorphine.^1^

We will discuss (1) how commonly the interviewees engaged in non-prescribed use of methadone and buprenorphine before entering treatment, (2) the various motives for use and whether there are differences between the cities, and (3) which sources the interviewees have used to get hold of the drugs and whether there are differences between the cities. The results are analyzed against an extensive review of previous research on non-prescribed use.

For many years, the access to OST in Sweden was severely restricted, but availability increased significantly following the launch of buprenorphine (Subutex®) in January 2000, from under 600 patients nationwide to almost 4000 patients in 2013 [21, 22]. This coincided with a sizeable increase in the number of methadone- and buprenorphine-related cases of death. While there were 9 deaths in 1998 where a forensic autopsy found traces of methadone or buprenorphine in the deceased’s body, the number increased to 201 in 2013 (the number of cases where a narcotic substance of some kind was detected rose from 286 to 748 during the same period) [23]. A study of methadone- and buprenorphine-related deaths found that only 20% of the deceased had had a prescription for these substances. It is therefore important to examine the non-prescribed use of these substances.

We focus on the geographical aspect because there is a lack of research that compares similarities and differences of non-prescribed use in different contexts. The drug situation and the availability of OST differ somewhat between the studied cities. This is discussed in greater detail in the “Methods” section.

The combination drug buprenorphine/naloxone (Suboxone®) was introduced in Sweden in November 2006 and has been used to varying degrees as complementing or replacing buprenorphine in the five cities that this study was conducted in. We distinguish between buprenorphine and buprenorphine/naloxone where this is relevant.

## Previous research

Non-prescribed use of methadone and buprenorphine is not a large international research area, but there is a growing body of research—especially in the US, Australia, and several western European countries—where the prevalence of non-prescribed use has been examined in different contexts and in different groups of drug users. The motives for this use have also been explored in several studies, while there has been less research on the sources that these drugs come from. Non-prescribed use of methadone and buprenorphine has received little research attention in Sweden or the other Nordic countries.

### Prevalence of non-prescribed use

Non-prescribed use of methadone began to gain attention at the beginning of the 1970s after the treatment had spread throughout the country [24, 25]. Ethnographic studies described how methadone had become integrated into the drug scenes, serving as a street drug among heroin users [26–28]. Similar experiences have emerged in many countries where methadone-based OST has been widely used [29–32].

Buprenorphine appeared on the illegal drug market in the 1980s in the form of Temgesic® painkillers [33], but it was only in the 1990s when it was launched as a medication (Subutex) in OST that buprenorphine became more established among users of illicit drugs [6]. During the first decade of the 2000s, it became increasingly common in many countries among injecting drug users and those with heroin dependence [6, 34, 35]. Finland has been one of the most conspicuous examples in this respect, as buprenorphine has been the predominant opioid on the Finnish illegal drug market. Two studies showed in 2007 that buprenorphine was the most common drug among injecting drug users in Helsinki and that 97% of those who sought OST had buprenorphine as their main drug [36, 37].

Studies on different groups of drug users show variation in the use of methadone and buprenorphine. The lifetime prevalence of non-prescribed use of methadone has varied between 17 and 95% [29, 30, 38–42], while the lifetime prevalence of non-prescribed use of buprenorphine has varied between 8 and 76% [37, 43–48]. The higher prevalence rates are found in studies focusing on individuals whose drug use is dominated by opioids [6, 34, 45]. The highest prevalence numbers—in terms of both lifetime and current use—is found in studies on those who inject heroin or other opioids and in studies on individuals who have sought treatment for opioid dependence [30, 37, 40, 41, 49]. A case in point is a Swedish study of non-prescribed use of buprenorphine conducted with 350 interviewees at a needle exchange program in Malmö in 2004. Almost half of the respondents had heroin as their main drug, and as many as 89% of these said that they had used buprenorphine during the past year. Among those whose main drug was amphetamine, the figure was 24% [44].

That the non-prescribed use varies from one place and time to another may depend on shifting availability and price of heroin and other opioids but also on the availability and setup of OST [6, 35, 50]. Greater access to OST can increase the supply of methadone and buprenorphine on the illegal market as a result of drug diversion (“leakage”) by the patients. However, greater availability can also decrease the illicit demand for these substances, when more people turn toward treatment [6, 51, 52]. Prior to this study, there are no comparative studies on differences in use between different locations.

Much of the research has focused on experienced drug users, most of all on individuals who have a long-term opioid dependence and/or who inject drugs. The results suggest that methadone and buprenorphine tend to come in late in a person’s drug use trajectory and that they are rarely the users’ primary choice within the opioid group [48, 53]. Some results go against these findings, however, at least regarding buprenorphine. The Finnish experiences should be mentioned here (see above), as well as some studies from other countries. In a survey among injecting drug users in the country of Georgia, 11.5% of the respondents said that buprenorphine was the drug that they first developed a dependence of [54], while a study from India suggested that drug users who had turned to injecting were more prone to using buprenorphine than heroin [55]. These studies, too, focus on experienced drug users.

Few studies have examined the use of methadone and buprenorphine among younger and/or recreational drug users. A study of ours in Sweden, drawing on register data and interviews with professionals, shows that these substances are very rare among adolescents and young adults who do not already have severe drug problems. We compared three different populations in the study: a general group of grade two high school students (16–17 years of age), a group that had sought help or had been referred to clinics for adolescents and young adults with problems related to alcohol or other drugs, and a group of young adults in care for severe drug problems. Both the register data and the interviews showed that methadone and buprenorphine were generally unknown among high school students and extremely uncommon among clients at outpatient clinics for adolescents. The substances were relatively common among young adults with serious drug problems and were often part of polydrug use [56].

### Motives for non-prescribed use

The fact that non-prescribed methadone and buprenorphine are mainly used by experienced drug users and by those with an established opioid dependence also shows in the motives driving the use. These are often related to the so-called pseudo-therapeutic reasons, which correlate with the intended medical use of these substances. Pseudo-therapeutic use can aim to mitigate or avoid withdrawal symptoms (“to stay straight”), to take care of one’s own detoxification, to stay clean from heroin, or to alleviate pain [6, 30, 34, 49, 50, 56–58]. In the study referred to above at the needle exchange program in Malmö, 87% of those whose main drug was heroin said that they had use non-prescribed buprenorphine for withdrawal symptoms or detoxification [44].

Another common motive for using non-prescribed methadone or buprenorphine is taking care of one’s own OST. The substances are here used as in regular treatment; roughly equal amounts are taken daily for an extended period, with the primary aim of staying away from other opioids. This kind of use has been documented in several studies [49, 51, 59–61].

Barriers to OST have been pointed out as crucially contributing to pseudo-therapeutic use [59, 60]. In a previous study, we interviewed 27 people who had treated themselves with methadone or buprenorphine for at least 3 months. They had started self-medicating because they wished to change their lives or to cut back on heroin but also because they felt there were barriers to OST. The barriers had to do with difficulties accessing treatment (because of strict admission criteria, limited availability, or a bureaucratic and demanding assessment process), difficulties remaining in treatment (as a result of being involuntarily discharged for relapsing), and ambivalence toward or reluctance to seek treatment (most of all as a result of a fear of stigmatization or disciplinary action) [51].

Non-prescribed methadone and buprenorphine are also used for euphoric purposes (“to get high”), either on their own or combined with other substances. This is a less common motive, at least in studies with experienced drug users whose main drug is heroin or other opioids [6, 30, 42]. However, getting high on buprenorphine is common in countries where it has become the predominant illicit opioid [62, 63].

Economic motives are also claimed to play a role in the non-prescribed use of methadone and buprenorphine, which are often available at an attractive price on the illegal market compared to heroin [7, 35, 42, 49]. Methadone and buprenorphine also have a demand among patients undergoing OST, when they have failed to pick up their doses or are unhappy with their prescribed dose [64, 65].

The route of administration of non-prescribed use varies. There are many who take the substances as intended medically, that is, orally in the case of methadone and sublingually (to dissolve under the tongue) with buprenorphine. Injecting methadone carries high risks but is done both within and outside of treatment [66, 67]. In an Australian study with 312 heroin users, 52% had at some point injected methadone, and 29% had done so during the past 6 months [68]. Buprenorphine is both injected and snorted relatively commonly [6, 30, 34, 44]. Buprenorphine is generally much less typically injected than heroin [34], but there are exceptions. In a Finnish study, 81% of those seeking treatment who had buprenorphine as their primary drug said that they mainly injected this drug. Among those who primarily used heroin and sought treatment, the corresponding figure was 70% [63].

To reduce the risk of improper use, buprenorphine is often prescribed in a buprenorphine/naloxone combination. Naloxone has been added to make injecting and snorting less appealing.^2^ Many studies have shown that buprenorphine/naloxone is injected and snorted to a much lesser extent than plain buprenorphine [6, 34, 62, 69, 70] and that it is less sought after on the illegal market [71, 72].

Different routes of administration can relate to different motives for use, as well as the availability of different formulations. Oral administration of methadone and sublingual use of buprenorphine are more common in pseudo-therapeutic use, while the users more commonly resort to injecting when they wish to get high [19, 46, 47]. There can also be other reasons for injecting: for example, withdrawal symptoms are alleviated more quickly if the drugs are injected. Injecting is also the most economic route of administration, because the bioavailability is at its greatest at injecting (the substances are absorbed more effectively by the body) [47]. In addition, for some people who have injected for a long time, the very ritual of injecting can be closely associated with pleasure and make the habit extremely hard to break. This is known as the needle fixation [73, 74].

### Sources of non-prescribed substances

Where do users of non-prescribed methadone or buprenorphine get hold of the substances? There is not much research on this, but the issue has been examined in several studies since the 1970s. The substances are typically said to come from friends, acquaintances, or family members, who share the drugs among themselves or sell them out of their own legal prescription [6, 32, 45, 64, 71, 75]. These persons are most often undergoing OST [32, 50, 64, 71], but there are also those who have been prescribed methadone or buprenorphine to relieve pain [76, 77]. The drugs are handed over after payment or as a gift in a relationship that builds on favors or friendship [32, 48, 71, 77]. Bourgois argues that a “moral economy of sharing” often emerges among drug users; in this norm system, it is considered morally wrong not to share among friends suffering from withdrawal symptoms [78].

Research on the diversion of methadone and buprenorphine by patients in OST is contradictory about the extent of the phenomenon [32, 64, 67, 79, 80]. While many studies speak for a low prevalence of such drug diversion, this is at odds with the often extremely high prevalence rate reported in studies of non-prescribed use. A previous study of ours suggests in fact that drug diversion may be considerably more common than has been assumed.^3^ In this study, 68% of the patients said that they had at some point sold or shared their drugs, and 24% declared having done so during the past month [72]. For a clear majority, diversion takes place to a limited extent and only sporadically. Only a small group does it more systematically [64, 81].

Dealers are another major source of non-prescribed substances [77]. Dealers in this context refer to people who sell pharmaceuticals that they do not have a prescription for (they may of course have got hold of the substances via patients who have acquired them on prescription). Dealers have been declared as the most common source of buprenorphine in a few studies [48, 61], but existing research has overall found dealers to be a less common source than friends, acquaintances, and family members [32, 45, 50, 64, 71, 75].

International smuggling of buprenorphine has been documented, especially between European countries. France and the Baltic countries have often been identified as countries of origin for buprenorphine sold at the illegal drug markets in Finland and Sweden [82, 83]. A study conducted by The National Board of Health and Welfare in Sweden on the buprenorphine seized by the Swedish police found that the countries of origin could be traced in more than half of the seizures. Over 90% of the traced tablets came from abroad, mainly from France [84]. However, we need to be very cautious in drawing conclusions based on data on seizures, because this data totally depends on the data collection, that is, police efforts [85]. There is a reason to assume that the police do not prioritize measures against OST patients’ dealing and sharing their medication with friends and acquaintances. Based on the study by The National Board of Health and Welfare, it is reasonable to conclude that illegal trafficking represents a not insignificant share of the illegal supply of buprenorphine in Sweden.

Frivolous or dubious prescriptions by doctors are another source identified in previous research. Such prescribing partly reflects how prescriptions are regulated in different countries [77]. For example, some research suggests that private clinics prescribe larger methadone doses and keep a poorer control than do dependence clinics in the public sector [86]. “Doctor-shopping,” the practice of patients visiting multiple doctors to obtain multiple prescriptions, has been relatively common in countries which lack centrally regulated OST. In France, where buprenorphine is prescribed by general practitioners, 15–20% of the prescriptions have been calculated to be the result of doctor-shopping [87]. Buprenorphine was prescribed more liberally in Sweden in 2000–2005, before the rules were tightened by The National Board of Health and Welfare [21].^4^

Other sources, such as online purchases and thefts from healthcare facilities, have seldom been mentioned in the research on non-prescribed use of methadone and buprenorphine. Their role has been marginal in the few inquiries which have specifically studied these sources [48, 88]. This accords with a systematic overview and meta-analysis of sources of non-prescribed pharmaceuticals in general, where theft and online purchases are recognized as uncommon sources [77].

## Methods

### Sampling, participants, and recruitment

This study involves 411 patients recruited from 9 public and 2 private OST clinics in 5 cities in southern Sweden. Structured interviews were conducted between May and December 2012. The participants were required to have undergone OST for at least four weeks to be included. Table 1 shows the distribution of the data between the five cities.

**Table 1:** Number of interviews in the five cities

City	Number	Proportion (%)
Malmö	196	47.7
Göteborg	118	28.7
Lund	54	13.1
Norrköping	22	5.4
Jönköping	21	5.1
Total	411	100.0

The five cities were chosen for their varied drug scenes and availability of OST. In the neighboring cities of Lund and Malmö, heroin appeared on the illegal drug market in the 1970s, while Göteborg and Norrköping are more recent heroin cities (1990s). Opioid pharmaceuticals have dominated the drug scene in Jönköping (and later also in Norrköping), but heroin has been uncommon. OST with methadone was introduced in Lund and Malmö in the early 1990s, and methadone continues to be the predominant prescribed drug in these cities. In Göteborg, Jönköping, and Norrköping, OST with buprenorphine began at the beginning of the 2000s, whereas methadone began to be used later (as a result of new national guidelines). Buprenorphine has been the dominant OST drug in these cities but has been replaced by buprenorphine/naloxone in Jönköping and Norrköping after this substance was launched in Sweden in 2007. We have merged these two cities in the analyses to avoid creating groups which are too small. All five cities have had a relatively low availability of OST.

We used two different data collection methods: (1) on-site interviews conducted by the two researchers (Johnson and Richert) and three project assistants and (2) peer-to-peer interviews conducted by patients.

The on-site interviews (*n* = 280) were made in all five cities. Announcements were posted on the clinic walls a week or two before our arrival. Written information and booking sheets were left with a secretary. We then spent 2–10 working days at the clinics, conducting appointed interviews and recruiting more participants among the visitors. The interviews were conducted in private rooms.

The peer-to-peer-interviews (*n* = 131) were conducted in the two largest cities—Göteborg and Malmö—through “privileged access interviewing” [89–91] by nine specially trained patients. The interviewers, five women and four men, had a stable lifestyle and had large contact networks in different patient groups. They recruited interviewees from among their circles and conducted the interviews at various sites outside of the clinics—in homes, cafés, parks, etc. Before the data collection began, both the peer interviewers and the project assistants were trained on the contents of the interview itself and on the interviewing technique. We have discussed in detail the method of privileged access interviewing and the process of recruiting interviewers in a previous article [91].

Due to the nature of the recruitment procedure, a sophisticated non-response analysis is not possible, but on the group level, we have received information on patient numbers, gender, age, type of medication, average dose, and pick-up routines from the 11 clinics. The clinics had in all 1006 patients, which means that the 411 interviews represented 40.8% of the total population (minimum 24.3%, maximum 64.3%). Table 2 shows a comparison between the study population and the clinics’ total population. There are significant differences only in pick-up routines; our material has an overrepresentation of patients who pick up their medication on 5–7 days of the week (34.5% vs. 21.2% in the total population) and an underrepresentation of patients who come and get their medication once a week or more seldom (29.2% vs. 42.3%). This difference was to be expected, as most of the interviewees were recruited at the clinics.

**Table 2:** Non-response analysis

Variable	Study group (411)	All patients (1006)	*P* value (*χ*^2^ test)
Gender (man)	74.7% (307)	75.2% (757)	0.736
Age (mean)	39.36 (411)	39.85 (1006)	
Medication			
Methadone	53.3% (219)	51.6% (519)	0.628
Buprenorphine	27.3% (112)	28.7% (289)	
Buprenorphine/naloxone	19.5% (80)	19.7% (198)	
Dose (mg)			
Methadone	99.32 (219)	98.55 (517)	
Mono-buprenorphine	19.02 (112)	19.44 (289)	
Buprenorphine/naloxone	18.78 (80)	18.88 (198)	
Pick-up routines			
5–7 days/week	34.5% (142)	21.4% (215)	0.000
2–4 days/week	36.3% (149)	35.8% (360)	
More seldom	29.2% (120)	42.8% (431)	

### Interviewing

The interview procedure was the same irrespective of who did the interviewing. The participants were first told about the project and its aims orally and in writing. They were told that the study was confidential, that it would not have an impact on their treatment, and that they could at any point stop the interview. The participants could subsequently choose between a gift voucher for SEK 200 (about € 19) or a book, regardless of whether they completed the interview or not.

The interviews were conducted with a standardized questionnaire. The questionnaire had 106 closed-ended and 5 open-ended questions covering the following areas: demographic information, health, social situation, drug use (past and current), experiences of own non-prescribed use of substitution drugs, treatment experiences, current OST and ideas about this treatment, and views on and own experiences of diversion. The interviews lasted on average about 1 h.

### Variables and statistical analysis

The variables examined in this article build on the following questions:

1. *Medication:* “What is your prescribed OST medication?” Response options: (a) methadone, (b) buprenorphine (Subutex, Buprenotex), and (c) buprenorphine-naloxone (Suboxone).
2. *Length of treatment:* “How long have you been in OST this treatment episode?” Response options: number of months.
3. *Experience of involuntary discharge:* “Have you ever been discharged from OST against your will?” Response options: yes/no.
4. *Use of methadone outside treatment:* “Have you used methadone outside treatment?” Response options: (a) no, (b) yes, only drinkable methadone, (c) yes, only methadone tablets, and (d) yes, both drinkable methadone and tablets. The answers have been dichotomized for the analyses in this article (yes/no).
5. *Use of buprenorphine outside treatment:* “Have you used buprenorphine (Subutex, Buprenotex) outside treatment?” Response options: yes/no.
6. *Use of buprenorphine/naloxone outside treatment:* “Have you used buprenorphine/naloxone (Suboxone) outside treatment?” Response options: yes/no.
7. *Injecting methadone:* “Have you injected methadone outside treatment?” Response options: yes/no.
8. *Route of administration for buprenorphine or buprenorphine/naloxone:* “When you were using buprenorphine or buprenorphine/naloxone, how did you usually take it?” Response options: (a) under the tongue, (b) injecting, (c) snorting, (d) smoking, and (e) other (specify).
9. *Motives for use outside treatment:* “Why did you use methadone, buprenorphine, or buprenorphine/naloxone?” Response options: (a) own detoxification, (b) to avoid withdrawal, (c) own OST, (d) to get high, (e) hard to get hold of heroin, (f) wanted to stay clean from heroin, and (g) other reason (specify).
10. *Sources of drugs outside treatment:* “Where did you usually get hold of methadone or buprenorphine?” Response options: (a) purchased from someone undergoing OST, (b) purchased from a dealer, (c) purchased from a patient in treatment other than OST, (d) from a doctor, from within health care, (e) online trade, and (f) another source (specify).
11. *Street price:* “Do you know the current street price for methadone, buprenorphine, or buprenorphine/naloxone?” The price was cited as per bottle (90 mg) of methadone and tablet (8 mg) of buprenorphine and buprenorphine/naloxone.

The data have been analyzed by frequency and average calculations and cross-tabulations. Chi-squared tests and variance analysis (ANOVA) were performed to establish a level of significance of group differences. All analyses were done in SPSS version 24 for Windows.

## Results

### Prevalence of non-prescribed use in the study population

Table 3 shows the prevalence of non-prescribed use for the three substances in the study population according to demographic variables, treatment variables, geographical location, and type of interviewer. The use of methadone (87.8%) and buprenorphine (80.5%) is very common in the population, while the use of buprenorphine/naloxone (50.6%) is not quite as common. This is probably because buprenorphine/naloxone had only been used for a few years in Sweden at the time of the interviews and that it was not prescribed at the clinics to a similar extent as methadone and buprenorphine. There are no clear differences in the use according to the demographic variables (gender, country of birth, and education).

**Table 3:** Non-prescribed use of methadone, buprenorphine, and buprenorphine/naloxone in the study population

	Non-prescribed use of methadone	Non-prescribed use of buprenorphine	Non-prescribed use of buprenorphine/naloxone
Total			
Whole population (411)	87.8%	80.5%	50.6%
Gender			
Woman (104)	85.6%	75.0%	54.1%
Man (307)	88.6%	82.4%	43.0%
Chi-2 (*P* value)	0.415	0.099	0.054
Country of birth			
Sweden (333)	88.9%	82.6%	53.5%
Other country (78)	83.3%	71.8%	42.1%
Chi-2 (*P* value)	0.177	0.030	0.073
Education			
Comprehensive school (195)	86.7%	82.6%	52.9%
High school or more (216)	88.9%	78.7%	50.0%
Chi-2 (*P* value)	0.491	0.324	0.563
Drug			
Methadone (219)	92.7%	74.0%	45.1%
Buprenorphine (112)	81.3%	87.5%	49.5%
Buprenorphine/naloxone (80)	83.8%	88.8%	70.9%
Chi-2 (*P* value)	0.005	0.002	0.000
Treatment period, length of			
Up to 1 year (138)	90.6%	88.4%	65.9%
1–3 years (140)	90.7%	85.7%	58.6%
More than 3 years (130)	82.3%	66.2%	25.8%
Chi-2 (*P* value)	0.054	0.000	0.000
Experience of involuntary discharge			
No (262)	85.9%	80.2%	52.3%
Yes (149)	91.3%	81.2%	49.7%
Chi-2 (*P* value)	0.108	0.795	0.606
City			
Malmö (196)	92.9%	75.5%	51.5%
Göteborg (118)	81.4%	88.1%	49.1%
Lund (54)	88.9%	77.8%	46.3%
Jönköping/Norrköping (43)	81.4%	86.0%	63.4%
Chi-2 (P value)	0.012	0.035	0.366
Interviewers			
Researchers (280)	88.2%	78.6%	49.1%
Privileged access interviewers (131)	87.0%	84.7%	56.2%
Chi-2 (*P* value)	0.731	0.142	0.184

Regarding the treatment variables, a greater share of patients had used those substances that they were later prescribed in OST. This is especially clear in the use of buprenorphine/naloxone. The differences may be down to the patients’ positive experiences of non-prescribed use of a certain substance; they may have asked to be given this substance at the beginning of the treatment. It might also be that patients who had been involuntarily discharged from a treatment program had continued to use the substance that they used to be prescribed and were now waiting to be readmitted into treatment.^5^ There are significant differences also in terms of the treatment period; patients who had been in ongoing treatment for longer than 3 years have less experience of buprenorphine and considerably less experience of buprenorphine/naloxone. The most likely explanation is that some patients in this group have not had the opportunity to use these substances outside treatment, because they were not available before the patients began their OST. As regards the experience of involuntary discharge, there are no differences. This suggests that the decision to seek OST is generally preceded by the non-prescribed use of methadone, buprenorphine, and buprenorphine/naloxone.

The use of methadone and buprenorphine varies between the cities, while the use of buprenorphine/naloxone seems to be more stable. In Malmö and Lund, methadone was the drug most commonly used without prescription, whereas buprenorphine was the most common drug in Göteborg and Jönköping/Norrköping. In a previous article, we showed that privileged access interviewing may produce different results than traditional researcher interviews, especially when it comes to sensitive topics. Since researchers conducted interviews in all cities, and privileged access interviewers only in Göteborg and Malmö, we have included the type of interviewer as a variable in Table 3. The analysis indicates that the differences between cities cannot be explained by the differing data collection methods. However, the differences correlate with the predominant drug prescribed in OST in the cities under study—methadone in Malmö and Lund, buprenorphine in Göteborg, and buprenorphine and later buprenorphine/naloxone in Jönköping/Norrköping.

### Motives for non-prescribed use in the five cities, and routes of administration

The interviewees’ declared motives for use are shown in Table 4, presented in relation to substance and city (many interviewees stated more than one motive). A general pattern is that many interviewees cited several different motives for their use. A clear majority said that they had used the drugs to avoid withdrawal symptoms; this was the most common response for all three substances (and many expressed it as having used the drugs to “stay straight”). Other pseudo-therapeutic motives were similarly common, such as using the drugs for own OST or to stay clean from heroin. Lack of heroin was a common motive for using methadone, but slightly less common for using buprenorphine and buprenorphine/naloxone. Using these substances for euphoric purposes (to “get high”) was the least common motive. Around a third had used methadone and buprenorphine in order to get high, but the proportion was lower for buprenorphine/naloxone (17.8%).^6^

**Table 4:** Motives for non-prescribed use in different cities

	Malmö	Göteborg	Lund	Jönköping/Norrköping	All cities	Chi-2 (*P* value)
Motives for use of methadone	(182)	(96)	(48)	(35)	(361)	
Avoid withdrawal symptoms	92.9	89.6	79.2	85.7	89.5	0.043
Lack of heroin	57.1	54.2	43.8	51.4	54.0	0.416
Own detoxification	64.3	40.6	43.8	45.7	53.5	0.001
Wanted to stay clean from heroin	56.6	50.0	58.3	28.6	52.4	0.017
Own substitution treatment	42.3	21.9	54.2	34.3	37.7	0.001
Get high	24.7	32.3	18.8	60.0	29.4	0.000
Motives for use of buprenorphine	(148)	(104)	(41)	(37)	(330)	
Avoid withdrawal	83.8	83.7	85.4	89.2	84.5	0.859
Lack of heroin	44.9	45.2	36.6	48.6	44.4	0.723
Own detoxification	53.4	45.2	61.0	54.1	51.8	0.331
Wanted to stay clean from heroin	43.9	52.9	61.0	54.1	50.0	0.191
Own substitution treatment	25.7	44.2	61.0	75.7	41.5	0.000
Get high	35.1	26.0	17.1	45.9	31.2	0.019
Motives for use of buprenorphine/naloxone	(100)	(57)	(25)	(26)	(208)	
Avoid withdrawal	77.0	82.5	76.0	69.2	77.4	0.601
Lack of heroin	39.0	45.6	36.0	26.9	38.9	0.434
Own detoxification	49.0	41.1	40.0	34.6	44.0	0.512
Wanted to stay clean from heroin	40.0	38.6	48.0	34.6	39.9	0.795
Own substitution treatment	25.0	31.6	40.0	50.0	31.7	0.076
Get high	17.0	14.0	20.0	26.9	17.8	0.540

Another pattern is that the various motives are cited to a lesser degree for buprenorphine/naloxone than for methadone and buprenorphine. This indicates that buprenorphine/naloxone is a less popular substance for non-prescribed use. Many interviewees declared spontaneously that they had used buprenorphine/naloxone when they had failed to get hold of buprenorphine (this response option was missing from the questionnaire).

There are significant differences between the cities in certain purposes of use, but the differences vary between the substances and are therefore hard to explain. In the case of own OST, different drugs (methadone or buprenorphine) seem to have been prioritized in different cities, but it is difficult to say why. The motive of getting high was cited more often by interviewees in Jönköping/Norrköping. This might be because synthetic opioids have had a more prominent role on the drug markets in these cities, while heroin has been available more sporadically. The general impression is however that there are no great differences in the declared motives in the different cities.

The interviewees’ routes of administration are shown in Table 5 for the whole population (some respondents had several routes of administration). The prescribed routes of administration were the most common for all three substances. Just under half of methadone users said that they had at some point injected the drug. It was more common to snort buprenorphine and buprenorphine/naloxone than to inject them. Buprenorphine/naloxone was most often administered sublingually, while injecting was uncommon. Many of those who had injected buprenorphine/naloxone said that the effect of the naloxone had made it a negative experience.

**Table 5:** Route of administration for non-prescribed use

Route of administration	Methadone (361)	Buprenorphine (330)	Buprenorphine/naloxone (208)
Sublingually	NV	79.7%	85.6%
Snorted	NV	70.6%%	51.0%
Injected	47.4%	40.9%	15.4%
Orally	100.0%	NA	NA

*NA* not asked, *NV* non-viable means of consumption

### Sourcing non-prescribed drugs in different cities

The sources given by the interviewees for methadone and buprenorphine (which here also includes buprenorphine/naloxone) are presented in Table 6. The results show clearly that patients undergoing OST were the most commonly cited source. This applies to both methadone and buprenorphine and to all cities, except for buprenorphine among interviewees in Göteborg (where dealers were the most common source).

**Table 6:** Sources for non-prescribed drugs in different cities

	Malmö	Göteborg	Lund	Jönköping/Norrköping	All cities	Chi-2 (*P* value)
Sources: methadone	(182)	(93)	(48)	(35)	(358)	
Patient in substitution treatment	74.2	71.0	66.7	65.7	71.5	0.620
Dealer	47.3	65.6	25.0	51.4	49.4	0.000
Other healthcare patient	12.1	10.8	6.3	34.3	13.1	0.001
From doctor/health care	6.0	0.0	4.2	2.9	3.9	0.107
Online	0.0	2.2	0.0	2.9	0.8	0.133
Other source	56.4	30.1	52.1	17.1	44.6	0.000
Sources: buprenorphine and buprenorphine/naloxone	(143)	(95)	(39)	(36)	(313)	
Patient in substitution treatment	75.5	56.8	76.9	72.2	69.6	0.013
Dealer	48.3	71.6	66.7	66.7	59.7	0.002
Other healthcare patient	6.3	0.0	0.0	5.6	3.5	0.036
From doctor/health care	3.5	3.2	2.6	0.0	2.9	0.728
Online	0.0	1.1	10.3	11.1	2.9	0.000
Other source	4.2	14.1	5.1	16.7	8.7	0.015
Street price						(ANOVA)
Methadone 90 mg	227	319	242	344	262	0.000
Buprenorphine 8 mg	103	155	108	271	137	0.000
Buprenorphine/naloxone 8 mg	84	127	93	215	112	0.000

Overall, dealers were the second most cited source for both methadone and buprenorphine, with interesting and significant differences between the cities. Dealers were the declared source of methadone to a much higher degree in Göteborg than in Lund, while in Malmö, the dealers were declared to be a source for buprenorphine less often than in the other cities. Such differences suggest considerable variation between the local drug markets in terms of the availability of illicit drugs and pharmaceuticals.

“Other sources” were cited by almost half of the interviewees in the case of methadone. This category consisted almost exclusively of people who said that they had purchased methadone from Danish patients and/or dealers in Denmark, smuggling small amounts for personal use. Denmark also appears as a source among a great many respondents in Göteborg but to a much smaller extent in Jönköping/Norrköping.^7^ There was no corresponding cross-border trade for buprenorphine, which has traditionally not been much used in Danish OST [92]. It also turned out that some users had bought buprenorphine in Stockholm and that this was more common among interviewees in Göteborg and Jönköping/Norrköping, which are geographically closer to Stockholm.

Other sources—other healthcare patients, doctors/health care, and online trade—were generally uncommon. Certain significant differences emerge between the cities, but the proportion of yes/no responses is so small that the differences are hard to explain. However, the proportion of those who said that they had acquired methadone from another healthcare patient was considerably bigger in Jönköping/Norrköping than in the other cities. This may be linked to what has already been referred to that synthetic opioids have cornered the drug markets in these cities.^8^

Table 6 also lists the average price for illegal purchases of methadone, buprenorphine, and buprenorphine/naloxone in the different cities. The prices varied greatly for all substances but were generally lowest in Malmö and Lund and highest in Jönköping/Norrköping. Buprenorphine had a higher street price in all cities than buprenorphine/naloxone.

## Discussion

Our study confirms many of the findings of previous research on the non-prescribed use of methadone and buprenorphine. Such use is very common among those whose drug use is dominated by opioids and especially among those who have sought treatment for opioid dependence. The prevalence rates of non-prescribed use in our study—87.8% for methadone, 80.5% for buprenorphine, and 50.6% for buprenorphine/naloxone—are high but correspond to those presented by previous studies among those who have sought OST [30, 38, 42, 43, 45].

Similarly, the motives given by the interviewees for non-prescribed use correspond to those named in previous research. The dominant motives pertain to what we have labeled as pseudo-therapeutic reasons, that is, those that align with the intended medical use of these substances [6, 30, 34, 44, 49, 50, 56–61]. It is often the case that the users want to avoid withdrawal symptoms, stay clean from heroin, detox themselves, or take care of their own OST by illicitly obtained drugs. The least common motive was non-prescribed use for euphoric purposes (to get high), but this too was reported by a sizeable minority of about 30% of those who had used non-prescribed methadone or buprenorphine.

Non-prescribed use of methadone and buprenorphine can carry considerable health risks, as we pointed out at the outset. Whether such use should be seen as a serious problem or not depends on how widespread the phenomenon is, but is also connected to which groups use these drugs and for which purposes. Pseudo-therapeutic use among people with an established opioid dependence can also lead to health benefits, which has been raised by many researchers [6, 19, 20, 51, 93]. Such use can therefore be seen as less serious than if the substances are used for euphoric purposes, by adolescents and young adults early in their drug-using trajectory [56]. Still, the positive aspects of non-prescribed use must not be read as reducing the problems and risks involved in such use.

Our study also by and large confirms the results of previous research as regards the sources for non-prescribed drugs. The methadone and buprenorphine markets have a structure which differs from that dealing with such illegal drugs as heroin, cocaine, or cannabis. The latter markets often draw on several bigger players who organize networks of dealers on different levels. The methadone and buprenorphine markets are based on substances originating from pharmaceutical companies. These markets are more decentralized, with many players selling smaller quantities of drugs that they have themselves been prescribed [28, 64, 76, 94, 95]. These are comparatively closed systems of current opioid users and patients undergoing OST [39, 64, 96]—a reasonable description also for the small-scale cross-border smuggling of methadone that takes place in southern Sweden. At the same time, however, a parallel and a more traditional illegal trade goes on with smuggled buprenorphine [82, 84].

The diversion of prescription drugs has been referred to as a “disorganized for-profit industry” with many kinds of actors [97]. This description is only partially applicable to the methadone and buprenorphine market. As we have shown previously, many of the drugs are handed over free of charge between family members, friends, and acquaintances with similar drug experiences [81, 96]. In the drug scenes that these people know and spend their time in, it is often considered morally right to share one’s drugs with those who do not have them at that point and thus risk getting “sick” (with withdrawal symptoms). Drugs may also be sold of course, but it is more often the case that there is an expectation that the person will do a similar favor later, if needed. It is this norm system that Bourgois calls a “moral economy of sharing” [78].

Our study is the first to examine geographical variations in non-prescribed use. Even if there are some significant disparities between the studied cities, the similarities outweigh the differences. The prevalence of non-prescribed use is very high in all cities, and the predominant motives are pseudo-therapeutic by their nature. This may be because all five cities have a history of insufficient availability of OST. The Swedish model for OST has been called restrictive and oriented toward rehabilitation, placing heavy demands on the patients and exercising heavy control [21, 98, 99].^9^ There has been a policy in all studied cities of discharging patients for repeated relapse, which is likely to be an important reason why many interviewees said that they had used the drugs for their own OST and detoxification and to stay clean from heroin. The fact that a low availability of OST can lead to a high illicit demand for methadone and buprenorphine has also been stressed in previous research [51, 61, 65, 100].

The Swedish model has gradually changed during the past decade. More treatment is now available, and it has become more oriented toward harm reduction [101]. These developments had begun at the time when the interviews were conducted, but much of the non-prescribed use examined here has taken place during the previous, more restrictive treatment model.

The geographical comparisons suggest that the drugs prescribed in OST have a great impact on which substances are used outside the treatment. This is not surprising. In Malmö and Lund, where methadone has long been the most common drug in OST, it is also the drug which is the most common substance in non-prescribed use. In Göteborg, Jönköping, and Norrköping, that drug is buprenorphine.

There are also big similarities between the cities in terms of the sources for the drugs. In all cities, patients undergoing OST were the source most often named by the interviewees—with one exception, there were more respondents in Göteborg for whom dealers were the most common source for buprenorphine. Dealers are relatively common sources in all cities. The main difference pertains to the proximity of the interviewees in Malmö and Lund to the Danish illegal market. Many respondents in the two cities said that they had purchased methadone from Danish dealers and patients, which we listed under “other source.”

As we pointed out in the research review, many previous studies have found that buprenorphine/naloxone has a lower abuse potential and a lower street price than drugs solely containing buprenorphine [6, 34, 62, 69–72, 102]. These results are also confirmed by our study. Many interviewees did say that they had used non-prescribed buprenorphine/naloxone, but it was less in demand and was available at a lower average price in all cities. The proportion of who said that they had injected or taken buprenorphine/naloxone intranasally was considerably lower than for buprenorphine. This indicates that buprenorphine/naloxone should be prioritized over buprenorphine when possible. Both the Swedish Medical Products Agency and The National Board of Health and Welfare have recommended buprenorphine/naloxone and have advised doctors against prescribing buprenorphine except in special cases, but these recommendations have been implemented in a checkered manner nationwide. In our study, it was only in Jönköping and Norrköping that the doctors clearly prioritized buprenorphine/naloxone over buprenorphine.

Another important implication that emerges from our study is that OST should be more readily available. Those who have an opioid dependence and request to be given these drugs should be able to obtain them as a part of a controlled medical treatment program [48, 61]. Even if Swedish OST has in recent years grown to be more inclusive, there are still many with an opioid dependence who involuntarily remain outside the treatment system. There are regional differences in this respect, and in some parts of the country, the queues are exceedingly long. Measures to shorten these queues, to lower the threshold, and to increase retention in OST can curb the illicit demand for methadone and buprenorphine.

Controls are still needed in OST to reduce the risk of drug diversion. Well thought-out pick-up routines and supervised dosage are important features in this control [11, 81, 103]. As such controls entail intrusion and restrictions on patients’ lives, the control measures need to be carefully balanced. Misguided or altogether too strict measures mean worse treatment, can lead to lower retention, and may thus increase rather than decrease the diversion problems.

## Limitations

The data was collected 7 years ago. Since then, the access to OST has increased significantly in Malmö, but not in other cities included in the study. Increased access to OST may have an impact on the non-prescribed use of methadone and buprenorphine, for instance regarding pseudo-therapeutic motives.

Our study group consists of individuals undergoing OST, and the majority has a long history of heroin or other opioid use. As regards the prevalence of non-prescribed use of methadone and buprenorphine, the motives driving the use, and the sources for getting hold of the drugs, the results correspond to those attained in previous research on similar samples, but cannot be generalized to other groups.

Another limitation is that the interviews dealt with historical data and lifetime prevalence of drug use. In order to study the prevalence and frequency of current use, a different kind of sample is called for, consisting of current opioid users not undergoing OST.

## Conclusions

Experiences of non-prescribed use of methadone and buprenorphine are extremely common among individuals who have an opioid dependence and are undergoing OST in southern Sweden. Such use typically has pseudo-therapeutic motives but is less common for euphoric purposes. This means that insufficient access to OST is likely to be a major factor behind the illicit demand for these substances. At the same time, patients in OST are the most common source for illicit methadone and buprenorphine, although other sources are also named, such as dealers and international cross-border trade.

Increased access to OST might well reduce the illicit demand for methadone and buprenorphine. Supervised dosage and other control measures are however an important part of the treatment regime to prevent the diversion of drugs to persons not in treatment. Also, buprenorphine/naloxone should be prescribed rather than buprenorphine, because buprenorphine/naloxone has a lower abuse potential.

## Footnotes

1 Alternative concepts that appear in the research are illicit (or illegal) use and non-medical use, but these are less suited to our context. While non-prescribed use is illegal in Sweden, it is unnecessary here to emphasize this aspect, as it is not the focus of our analysis. The concept of non-medical use runs the risk of being misleading, as the non-prescribed use is often driven by medically related (pseudo-therapeutic) motives. We will return to this question later in this article.

2 If the drug is injected or snorted, naloxone has a high bioavailability and thus counteracts the effects of buprenorphine, which leads to an unpleasant sensation. Taken sublingually, naloxone has a very low bioavailability, and the combination drug works like regular buprenorphine. Buprenorphine/naloxone is available both as tablets and sublingual film. The film sticks onto the palate, which further makes inappropriate use more difficult.

3 Our previous study builds on the same data set as this article. To say that one has used methadone and buprenorphine outside treatment seems unproblematic for most drug users. The low figures on drug diversion however suggest a problem with such self-reported information. To tell a researcher that they have sold or shared their drugs with others may be sensitive, even if the interviewees trust the researcher’s promises of confidentiality. We dealt with this problem by two different data collection techniques, using interviews conducted by researchers and interviews conducted by trained patients through “privileged access interviewing,” and then comparing the results. The idea was that the likelihood would grow of the interviewees reporting sensitive information if they were interviewed by an insider with own experience of substitution treatment. The data collection techniques are described in more detail in the methods section.

4 In 2000–2005, all Swedish doctors with a prescriptive authority were entitled to prescribe high-dose buprenorphine (Subutex). The prescribing of methadone for substitution treatment was however strictly regulated by directives from The National Board of Health and Welfare and was only allowed for psychiatric specialists. As of 2005, the two substances have been regulated by the same directives, and buprenorphine has been regulated similarly to methadone. This was done to prevent overprescribing and drug diversion onto the illegal market.

5 Patients who had been involuntarily discharged were issued an obligatory ban preventing them from seeking substitution treatment again for a fixed period. This period, set by The National Board of Health and Welfare, was 6 months as of 2005, was reduced to 3 months in 2010, and was abolished in 2016.

6 We also asked the interviewees what their most common motives had been to use the different substances. “Avoiding withdrawal symptoms” was by far the most common motive with all substances (54.0% for methadone, 48.0% for buprenorphine, and 48.7% for buprenorphine/naloxone), “own substitution treatment” was the second most common motive (14.8% for methadone, 26.8% for buprenorphine, and 20.3% for buprenorphine/naloxone), and “own detoxification” was the third most common motive (9.8% for methadone, 7.9% for buprenorphine, and 8.6% for buprenorphine/naloxone). The ranking order for the most common motives was the same in all cities.

7 Copenhagen, the capital of Denmark, is close to both Lund and Malmö. A train ride from Malmö to Copenhagen via the Öresund Bridge takes about 30 min.

8 We also asked the interviewees about their most common source for the drugs. As regards methadone, patients undergoing substitution treatment were clearly the most common source (49.1%), followed by “other source” (that is, Denmark, 25.3%), and dealers (21.3%). Patients in substitution treatment were the most common source also for buprenorphine (56.4%), followed by dealers (35.5%), and “other source” (5.1%). Sources other than these were only marginally important.

9 From a European perspective, the Swedish model has been marked by strict admission criteria, government rules which have led to discharging patients for repeated relapse, and by a ban which has prevented the discharged patients from seeking admission into new treatment for a certain period.

## References

[1] Mattick, R. P, Breen, C, Kimber, J. & Davoli, M.. Methadone maintenance therapy versus no opioid replacement therapy for opioid dependence. Cochrane Database Syst Rev. 2009. 10.1002/14651858.CD002209 12519570

[2] Amato L, Minozzi S, Davoli M, Vecchi S. Psychosocial combined with agonist maintenance treatments versus agonist maintenance treatments alone for treatment of opioid dependence. Cochrane Database Syst Rev. 2011. 10.1002/14651858.CD004147.pub4 21975742

[3] Mattick RP, Breen C, Kimber J, Davoli M. Buprenorphine maintenance versus placebo or methadone maintenance for opioid dependence. Cochrane Database Syst Rev. 2014. 10.1002/14651858.CD002207.pub3 18425880

[4] Connery HS (2015). Medication-assisted treatment of opioid use disorder: review of the evidence and future directions. Harv Rev Psychiatry.

[5] Fugelstad A, Stenbacka M, Leifman A, Nylander M, Thiblin I (2007). Methadone maintenance treatment: the balance between life-saving treatment and fatal poisonings. Addiction.

[6] Yokell MA, Zaller ND, Green TC, Rich JD (2011). Buprenorphine and buprenorphine/naloxone diversion, misuse, and illicit use: an international review. Curr Drug Abuse Rev.

[7] Aitken CK, Higgs PG, Hellard ME (2008). Buprenorphine injection in Melbourne, Australia: an update. Drug Alcohol Rev.

[8] Reimer J, Wright N, Somaini L, Roncero C, Maremmani I, McKeganey N, Littlewood R, Krajci P, Alho H (2016). The impact of misuse and diversion of opioid substitution treatment medicines: evidence review and expert consensus. Eur Addict Res.

[9] Milroy CM, Forrest ARW (2000). Methadone deaths: a toxicological analysis. J Clin Pathol.

[10] Seymour A, Black M, Jay J, Cooper G, Weir C, Oliver J (2003). The role of methadone in drug-related deaths in the west of Scotland. Addiction.

[11] Morgan O, Griffiths C, Hickman M (2006). Association between availability of heroin and methadone and fatal poisoning in England and Wales 1993–2004. Int J Epidemiol.

[12] Madden ME, Shapiro SL (2011). The methadone epidemic: methadone-related deaths on the rise in Vermont. Am J Forensic Med Pathol.

[13] Auriacombe M, Fatséas M, Dubernet J, Daulouede JP, Tignol J (2004). French field experience with buprenorphine. Am J Addict.

[14] Mégarbane B, Hreiche R, Pirnay S, Marie N, Baud FJ (2006). Does high-dose buprenorphine cause respiratory depression?. Toxicol Rev.

[15] Seldén T, Ahlner J, Druid H, Kronstrand R (2012). Toxicological and pathological findings in a series of buprenorphine related deaths: possible risk factors for fatal outcome. Forensic Sci Int.

[16] Wikner BN, Öhman I, Seldén T, Druid H, Brandt L, Kieler H (2014). Opioid-related mortality and filled prescriptions for buprenorphine and methadone. Drug Alcohol Rev.

[17] Bernard JP, Havnes I, Slørdal L, Waal H, Mørland J, Khiabani HZ (2013). Methadone-related deaths in Norway. Forensic Sci Int.

[18] Ledberg A (2017). Mortality related to methadone maintenance treatment in Stockholm, Sweden, during 2006–2013. J Subst Abuse Treat.

[19] Harris M, Rhodes T (2013). Methadone diversion as a protective strategy: the harm reduction potential of ‘generous constraints’. Int J Drug Policy.

[20] Bridge TP, Fudala PJ, Herbert S, Leiderman DB (2003). Safety and health policy considerations related to the use of buprenorphine/naloxone as an office-based treatment for opiate dependence. Drug Alcohol Depend.

[21] Johnson B. After the storm: developments in maintenance treatment policy and practice in Sweden 1987–2006: Nordic Centre for Alcohol and Drug Research; 2007.

[22] Socialstyrelsen (2015). Kartläggning av verksamheter som bedriver läkemedelsassisterad behandling vid opiatberoende.

[23] Toxreg. Research database managed by Anna Fugelstad, Karolinska Institute.

[24] Walter PV, Sheridan BK, Chambers CD, Chambers CD, Brill L (1972). Methadone diversion: a study of illicit availability. Methadone: Experiences and Issues.

[25] Weppner RS, Stephens RC, Conrad HT (1972). Methadone: some aspects of its legal and illegal use. Am J Psychiatry.

[26] Goldman FR, Thistel CI (1978). Diversion of methadone: illicit methadone use among applicants to two metropolitan drug abuse programs. Int J Addict.

[27] Agar MH, Stephens RC (1975). The methadone street scene: the addict’s view. Psychiatry.

[28] Agar M (1977). Going through the changes: methadone in New York City. Human Org.

[29] Lauzon P, Vincelette J, Bruneau J, Lamothe F, Lachance N, Brabant M, Soto J (1994). Illicit use of methadone among IV drug users in Montreal. J Subst Abuse Treat.

[30] Roche A, McCabe S, Smyth BP (2008). Illicit methadone use and abuse in young people accessing treatment for opiate dependence. Eur Addict Res.

[31] Maremmani I, Pacini M, Pani PP, Popovic D, Romano A, Maremmani AG, Deltito J, Perugi G (2009). Use of street methadone in Italian heroin addicts presenting for opioid agonist treatment. J Addict Dis.

[32] Duffy P, Baldwin H (2012). The nature of methadone diversion in England: a Merseyside case study. Harm Reduct J.

[33] O’Connor JJ, Moloney E, Travers R, Campbell A (1988). Buprenorphine abuse among opiate addicts. British J Addict.

[34] Lofwall MR, Walsh SL (2014). A review of buprenorphine diversion and misuse: the current evidence base and experiences from around the world. J Addict Med.

[35] Mravčík V, Janíková B, Drbohlavová B, Popov P, Pirona A (2018). The complex relation between access to opioid agonist therapy and diversion of opioid medications: a case example of large-scale misuse of buprenorphine in the Czech Republic. Harm Reduct J.

[36] Aalto M, Halme J, Visapää JP, Salaspuro M (2007). Buprenorphine misuse in Finland. Subst Use Misuse.

[37] Alho H, Sinclair D, Vuori E, Holopainen A (2007). Abuse liability of buprenorphine–naloxone tablets in untreated IV drug users. Drug Alcohol Depend.

[38] Vlahov D, O’driscoll P, Mehta SH, Ompad DC, Gern R, Galai N, Kirk GD (2007). Risk factors for methadone outside treatment programs: Implications for HIV treatment among injection drug users. Addiction.

[39] Davis WR, Johnson BD (2008). Prescription opioid use, misuse, and diversion among street drug users in New York City. Drug Alcohol Depend.

[40] Ompad DC, Fuller CM, Chan CA, Frye V, Vlahov D, Galea S (2008). Correlates of illicit methadone use in New York City: a cross-sectional study. BMC Public Health.

[41] Hall MT, Leukefeld CG, Havens JR (2013). Factors associated with high-frequency illicit methadone use among rural Appalachian drug users. Am J Drug Alcohol Abuse.

[42] Schmidt CS, Schulte B, Wickert C, Thane K, Kuhn S, Verthein U, Reimer J (2013). Non-prescribed use of substitution medication among German drug users: prevalence, motives and availability. Int J Drug Policy.

[43] Jenkinson RA, Clark NC, Fry CL, Dobbin M (2005). Buprenorphine diversion and injection in Melbourne, Australia: an emerging issue?. Addiction.

[44] Håkansson A, Medvedeo A, Andersson M, Berglund M (2007). Buprenorphine misuse among heroin and amphetamine users in Malmo, Sweden: purpose of misuse and route of administration. Eur Addict Res.

[45] Bazazi AR, Yokell M, Fu JJ, Rich JD, Zaller ND (2011). Illicit use of buprenorphine/naloxone among injecting and noninjecting opioid users. J Addict Med.

[46] Daniulaityte R, Falck R, Carlson RG (2012). Illicit use of buprenorphine in a community sample of young adult non-medical users of pharmaceutical opioids. Drug Alcohol Depend.

[47] Bretteville-Jensen AL, Lillehagen M, Gjersing L, Andreas JB (2015). Illicit use of opioid substitution drugs: prevalence, user characteristics, and the association with non-fatal overdoses. Drug Alcohol Depend.

[48] Cicero TJ, Ellis MS, Chilcoat HD (2018). Understanding the use of diverted buprenorphine. Drug Alcohol Depend.

[49] Schuman-Olivier Z, Albanese M, Nelson SE, Roland L, Puopolo F, Klinker L, Shaffer HJ (2010). Self-treatment: illicit buprenorphine use by opioid-dependent treatment seekers. J Subst Abuse Treat.

[50] Gwin Mitchell S, Kelly SM, Brown BS, Schacht Reisinger H, Peterson JA, Ruhf A, Agar MH, O’Grady KE, Schwartz RP (2009). Uses of diverted methadone and buprenorphine by opioid-addicted individuals in Baltimore, Maryland. Am J Addict.

[51] Richert T, Johnson B (2015). Long-term self-treatment with methadone or buprenorphine as a response to barriers to opioid substitution treatment: the case of Sweden. Harm Reduct J.

[52] Reddon H, Ho J, DeBeck K, Milloy MJ, Liu Y, Dong H, Ahamad K, Wood E, Kerr T, Hayashi K (2018). Increasing diversion of methadone in Vancouver, Canada, 2005–2015. J Subst Abuse Treat.

[53] Schulte B, Schmidt CS, Strada L, Götzke C, Hiller P, Fischer B, Reimer J (2016). Non-prescribed use of opioid substitution medication: Patterns and trends in sub-populations of opioid users in Germany. Int J Drug Policy.

[54] Otiashvili D, Zabransky T, Kirtadze I, Piralishvili G, Chavchanidze M, Miovsky M (2010). Why do the clients of Georgian needle exchange programmes inject buprenorphine?. Eur Addict Res.

[55] Solomon SS, Desai M, Srikrishnan AK, Thamburaj E, Vasudevan CK, Kumar MS, Solomon S, Celentano DD, Mehta SH (2010). The profile of injection drug users in Chennai, India: identification of risk behaviours and implications for interventions. Subst Use Misuse.

[56] Richert T, Johnson B (2013). Illicit use of methadone and buprenorphine among adolescents and young adults in Sweden. Harm Reduct J.

[57] Cicero TJ, Ellis MS, Surratt HL, Kurtz SP (2014). Factors contributing to the rise of buprenorphine misuse 2008–2013. Drug Alcohol Depend.

[58] Allen B, Harocopos A (2016). Non-prescribed buprenorphine in New York City: motivations for use, practices of diversion, and experiences of stigma. J Subst Abuse Treat.

[59] Peterson JA, Schwartz RP, Gwin Mitchell S, Reisinger HS, Kelly SM, O’Grady KE, Brown BS, Agar MH (2010). Why don’t out-of-treatment individuals enter methadone treatment programmes?. Int J Drug Policy.

[60] Stöver H (2011). Barriers to opioid substitution treatment access, entry and retention: a survey of opioid users, patients in treatment, and treating and non-treating physicians. Eur Addict Res.

[61] Lofwall MR, Havens JR (2012). Inability to access buprenorphine treatment as a risk factor for using diverted buprenorphine. Drug Alcohol Depend.

[62] Simojoki K, Alho H (2013). A five-year follow-up of buprenorphine abuse potential. J Alcoholism Drug Depend.

[63] Uosukainen H, Kauhanen J, Voutilainen S, Föhr J, Paasolainen M, Tiihonen J, Laitinen K, Onyeka IN, Bell JS (2013). Twelve-year trend in treatment seeking for buprenorphine abuse in Finland. Drug Alcohol Depend.

[64] Spunt B, Hunt DE, Lipton DS, Goldsmith DS (1986). Methadone diversion: A new look. J Drug Issues.

[65] Duffy P, Mackridge AJ (2014). Use and diversion of illicit methadone – under what circumstances does it occur, and potential risks associated with continued use of other substances. J Subst Use.

[66] Darke S, Topp L, Ross J (2002). The injection of methadone and benzodiazepines among Sydney injecting drug users 1996–2000: 5-year monitoring of trends from the Illicit Drug Reporting System. Drug Alcohol Rev.

[67] Winstock AR, Lea T, Sheridan J (2008). Prevalence of diversion and injection of methadone and buprenorphine among clients receiving opioid treatment at community pharmacies in New South Wales, Australia. Int J Drug Policy.

[68] Darke S, Ross J, Hall W (1996). Prevalence and correlates of the injection of methadone syrup in Sydney, Australia. Drug Alcohol Depend.

[69] Smirnov A, Kemp R (2012). Use and misuse of opioid replacement therapies: a Queensland study. Subst Use Misuse.

[70] Jones JD, Sullivan MA, Vosburg SK, Manubay JM, Mogali S, Metz V, Comer SD (2015). Abuse potential of intranasal buprenorphine versus buprenorphine/naloxone in buprenorphine-maintained heroin users. Addict Biol.

[71] Larance B, Degenhardt L, Lintzeris N, Bell J, Winstock A, Dietze P, Mattick R, Ali R, Horyniak D (2011). Post-marketing surveillance of buprenorphine-naloxone in Australia: diversion, injection and adherence with supervised dosing. Drug Alcohol Depend.

[72] Johnson B, Richert T (2015). Diversion of methadone and buprenorphine by patients in opioid substitution treatment in Sweden: prevalence estimates and risk factors. Int J Drug Policy.

[73] McBride AJ, Pates RM, Arnold K, Ball N (2001). Needle fixation, the drug user’s perspective: a qualitative study. Addiction.

[74] Horyniak D, Armstrong S, Higgs P, Wain D, Aitken C (2007). Poor man’s smack: a qualitative study of buprenorphine injecting in Melbourne, Australia. Contemp Drug Prob.

[75] Weppner RS, Stephens RC (1973). The illicit use and diversion of methadone on the street as related by hospitalized addicts. J Drug Issues.

[76] Cicero TJ, Inciardi JA (2005). Diversion and abuse of methadone prescribed for pain management. JAMA.

[77] Hulme S, Bright D, Nielsen S (2018). The source and diversion of pharmaceutical drugs for non-medical use: a systematic review and meta-analysis. Drug Alcohol Depend.

[78] Bourgois P (1998). The moral economies of homeless heroin addicts: confronting ethnography, HIV risk, and everyday violence in San Francisco shooting encampments. Subst Use Misuse.

[79] Dale-Perera A, Goulão J, Stöver H (2012). Quality of care provided to patients receiving opioid maintenance treatment in Europe: results from the EQUATOR analysis. Heroin Addict Relat Clin Prob.

[80] Launonen E, Alho H, Kotovirta E, Wallace I, Simojoki K (2015). Diversion of opioid maintenance treatment medications and predictors for diversion among Finnish maintenance treatment patients. Int J Drug Policy.

[81] Johnson B, Richert T (2015). Diversion of methadone and buprenorphine from opioid substitution treatment: patients who regularly sell or share their medication. J Addict Dis.

[82] Rönkä S, Virtanen A. Finland drug situation 2009: 2009 national report to the EMCDDA: new developments, trends and in-depth information on selected issues: Report/National Institute for Health and Welfare 45/2009; 2009.

[83] Alho H, D’Agnone O, Krajci P, McKeganey N, Maremmani I, Reimer J, Roncero C, Somaini L, Wright N, Littlewood R (2015). The extent of misuse and diversion of medication for agonist opioid treatment: a review and expert opinions. Heroin Addict Relat Clin Prob.

[84] Socialstyrelsen (2015). Buprenorfin och metadon på den illegala drogmarknaden.

[85] Sarnecki J (2014). Introduktion till kriminologi. Volym 1.

[86] Strang J, Hall W, Hickman M, Bird SM (2010). Impact of supervision of methadone consumption on deaths related to methadone overdose (1993-2008): analyses using OD4 index in England and Scotland. BMJ.

[87] Pradel V, Frauger E, Thirion X, Ronfle E, Lapierre V, Masut A, Coudert C, Blin O, Micallef J (2009). Impact of a prescription monitoring program on doctor-shopping for high dosage buprenorphine. Pharmacoepidemiol Drug Safety.

[88] Ng S, Macgregor S (2012). Pharmaceutical drug use among police detainees. Res Pract.

[89] Griffiths P, Gossop M, Powis B, Strang J (1993). Reaching hidden populations of drug users by privileged access interviewers: methodological and practical issues. Addiction.

[90] Kuebler D, Hausser D (1997). The Swiss Hidden Population Study: practical and methodological aspects of data collection by privileged access interviewers. Addiction.

[91] Johnson B, Richert T (2016). A comparison of privileged access interviewing and traditional interviewing methods when studying drug users in treatment. Addict Res Theory.

[92] Frank VA, Bjerge B, Houborg E (2013). Shifts in opioid substitution treatment policy in Denmark from 2000–2011. Subst Use Misuse.

[93] Havnes IA, Clausen T, Middelthon AL (2013). ‘Diversion’ of methadone or buprenorphine: ‘harm’ versus ‘helping’. Harm Reduct J.

[94] Fountain J, Strang J, Gossop M, Farrel M, Griffiths P (2000). Diversion of prescribed drugs by drug users in treatment: analysis of the UK market and new data from London. Addiction.

[95] Fountain J, Strang J, Tober G, Strang J (2003). The play, the plot and the players: the illicit market in methadone. Methadone Matters.

[96] Johnson B, Richert T (2015). Diversion of methadone and buprenorphine from opioid substitution treatment: the importance of patients’ attitudes and norms. J Subst Abuse Treat.

[97] Inciardi JA, Surratt HL, Kurtz SP, Cicero TJ (2007). Mechanisms of prescription drug diversion among drug-involved club-and street-based populations. Pain Med.

[98] Petersson FJ (2013). Excusing exclusion: accounting for rule-breaking and sanctions in a Swedish methadone clinic. Int J Drug Policy.

[99] Svensson B, Andersson M (2012). Involuntary discharge from medication-assisted treatment for people with heroin addiction–patients’ experiences and interpretations. Nordic Stud Alcohol Drugs.

[100] Carroll JJ, Rich JD, Green TC (2018). The more things change: buprenorphine/naloxone diversion continues while treatment remains inaccessible. J Addict Med.

[101] Andersson, L & Johnson B.. Patient choice as a means of empowerment in opioid substitution treatment: a case from Sweden. Drugs Educ Prevent Policy. (in press)

[102] Comer SD, Sullivan MA, Vosburg SK, Manubay J, Amass L, Cooper ZD, Saccone P, Kleber HD (2010). Abuse liability of intravenous buprenorphine/naloxone and buprenorphine alone in buprenorphine-maintained intravenous heroin abusers. Addiction.

[103] Strang J, Sheridan J (2001). Methadone prescribing to opiate addicts by private doctors: comparison with NHS practice in south east England. Addiction.

